# Supplementary material for: The Role of Vitamin D in the Development of Diabetes Post Gestational Diabetes Mellitus: A Systematic Literature Review
Source: Nutrients. 2020 Jun 10;12(6):1733. doi: 10.3390/nu12061733 (PMC7352830; doi:10.3390/nu12061733)
Supplement: Supplementary file 1 [file nutrients-12-01733-s001.pdf]

The role of vitamin D in the development of diabetes post gestational diabetes mellitus: a systematic literature review

A.C. Keller, C. Varela, R. Dangol, N.P. Damm, B.L. Heitmann, M. N. Händel

Supplementary materials

Contents

Table S1: List of excluded studies after full text screening and reasons for their exclusion............................ 2

Table S2: Baseline maternal mean (SD) BMI in the six included studies on vitamin D in gestational diabetes and markers of type 2 diabetes development.. 11

Table S3: Synthesis of results with direction of associations by outcome among the three included RCTs on vitamin D in gestational diabetes and markers of type 2 diabetes development 12

Table S4: Summary of ROBINS-I and Cochrane Risk of Bias assessment 14

The role of vitamin D in the development of diabetes post gestational diabetes mellitus: a systematic literature review

A.C. Keller, C. Varela, R. Dangol, N.P. Damm, B.L. Heitmann, M. N. Händel

---

Table S1: List of excluded studies after full text screening and reasons for their exclusion

1. Anastasiou E.; Athanasiadou A.; Vasileiou V.; Giakoumi A.; Papageorgiou G.; Zapanti E.; Alevizaki M.. Vitamin D deficiency and isolated fasting hyperglycaemia in pregnancy. Diabetologia 2010;53(SUPPL. 1):S438. [DOI: http://dx.doi.org/10.1007/s00125-010-1872-z]

   Reason for exclusion Wrong outcomes

2. Arora C.P.. Role of vitamin D in modulating gestational diabetes. Biopolymers and Cell 2011;27(2):85-92. [DOI:]

   Reason for exclusion Wrong study design

3. Asemi, Z; Hashemi, T; Karamali, M; Samimi, M; Esmaillzadeh, A. Effects of vitamin D supplementation on glucose metabolism, lipid concentrations, inflammation, and oxidative stress in gestational diabetes: a double-blind randomized controlled clinical trial1-3. 2013;98(6):1425-1432. [DOI: 10.3945/ajcn.113.072785]

   Reason for exclusion Wrong study design

4. Asemi, Z; Hashemi, T; Karamali, M; Samimi, M; Esmaillzadeh, A. Effects of vitamin D supplementation on glucose metabolism, lipid concentrations, inflammation, and oxidative stress in gestational diabetes: a double-blind randomized controlled clinical trial. 2013;98(6):1425-1432. [DOI: 10.3945/ajcn.113.072785]

   Reason for exclusion Wrong outcomes

5. Azzam E.Z.; El-Aghoury A.A.; Abd El-naby E.-S.E.; El-Maadawy S.A.. Studying the relation between vitamin D deficiency and glycemic state among pregnant women with gestational diabetes. Diabetes and Metabolic Syndrome: Clinical Research and Reviews 2019;13(2):1505-1509. [DOI: http://dx.doi.org/10.1016/j.dsx.2019.03.007]

   Reason for exclusion Wrong outcomes

6. Baker, A. M.; Haeri, S.; Camargo, C. A.; Stuebe, A. M.; Boggess, K. A.. First-trimester maternal vitamin D status and risk for gestational diabetes (GDM) a nested case-control study. Diabetes-Metabolism Research and Reviews 2012;28(2):164-168. [DOI: 10.1002/dmrr.1282]

   Reason for exclusion Wrong outcomes

7. Bal, M.; Ersoy, G. S.; Demirtas, O.; Kurt, S.; Tasyurt, A.. Vitamin D deficiency in pregnancy is not associated with diabetes mellitus development in pregnant women at low risk for gestational diabetes. Turkish Journal of Obstetrics and Gynecology 2016;13(1):23-26. [DOI: 10.4274/tjod.10170]

Reason for exclusion Wrong outcomes

8. Casey C.; McGinty A.; Holmes VA.; Hill AJ.; Patterson CC.; Young IS.; McCance DR.. Maternal vitamin D and markers of glycaemia during pregnancy in the Belfast centre of the Hyperglycaemia and Adverse Pregnancy Outcome study.. Diabetic medicine : a journal of the British Diabetic Association 2018;35(7):972-979. [DOI: 10.1111/dme.13632]

Reason for exclusion Wrong outcomes

9. Charatcharoenwitthaya N.; Somprasit C.; Chanthasenanont A.; Chailurkit L.-O.; Chanprasertyothin S.; Ongphiphadhanakul B.. Maternal Vitamin D levels, polymorphism of Vitamin D binding protein gene and risk for gestational diabetes mellitus. Endocrine Reviews 2014;35(SUPPL. 3). [DOI:]

Reason for exclusion Wrong outcomes

10. Cho, G. J.; Hong, S. C.; Oh, M. J.; Kim, H. J.. Vitamin D deficiency in gestational diabetes mellitus and the role of the placenta. American Journal of Obstetrics and Gynecology 2013;209(6). [DOI: 10.1016/j.ajog.2013.08.015]

Reason for exclusion Wrong outcomes

11. Christesen HT.; Falkenberg T.; Lamont RF.; Jørgensen JS.. The impact of vitamin D on pregnancy: a systematic review.. Acta obstetricia et gynecologica Scandinavica 2012;91(12):1357-67. [DOI: 10.1111/aogs.12000]

Reason for exclusion Wrong study design

12. Clifton-Bligh RJ.; McElduff P.; McElduff A.. Maternal vitamin D deficiency, ethnicity and gestational diabetes.. Diabetic medicine : a journal of the British Diabetic Association 2008;25(6):678-84. [DOI: 10.1111/j.1464-5491.2008.02422.x]

Reason for exclusion Wrong outcomes

13. Corcoy R.; Mendoza LC.; Simmons D.; Desoye G.; Adelantado JM.; Chico A.; Devlieger R.; van Assche A.; Galjaard S.; Timmerman D.; Lapolla A.; Dalfra MG.; Bertolotto A.; Harreiter J.; Wender-Ozegowska E.; Zawiejska A.; Kautzky-Willer A.; Dunne FP.; Damm P.; Mathiesen ER.; Jensen DM.; Andersen LLT.; Tanvig M.; Hill DJ.; Jelsma JG.; Snoek FJ.; Köfeler H.; Trötzmüller M.; Lips P.; van Poppel MNM.. The DALI vitamin D randomized controlled trial for gestational diabetes mellitus prevention: No major benefit shown besides vitamin D sufficiency.. Clinical nutrition (Edinburgh, Scotland) 2019. [DOI: 10.1016/j.clnu.2019.04.006]

Reason for exclusion Wrong patient population

14. De-Regil, LM; Palacios, C; Lombardo, LK; Peña-Rosas, JP. Vitamin D supplementation for women during pregnancy. Cochrane Database of Systematic Reviews 2016;(1). [DOI: 10.1002/14651858.CD008873.pub3]

    Reason for exclusion Wrong study design

15. Eggemoen ÅR.; Waage CW.; Sletner L.; Gulseth HL.; Birkeland KI.; Jenum AK.. Vitamin D, Gestational Diabetes, and Measures of Glucose Metabolism in a Population-Based Multiethnic Cohort.. Journal of diabetes research 2018;2018:8939235. [DOI: 10.1155/2018/8939235]

    Reason for exclusion Wrong outcomes

16. Farrant HJ.; Krishnaveni GV.; Hill JC.; Boucher BJ.; Fisher DJ.; Noonan K.; Osmond C.; Veena SR.; Fall CH.. Vitamin D insufficiency is common in Indian mothers but is not associated with gestational diabetes or variation in newborn size.. European journal of clinical nutrition 2009;63(5):646-52. [DOI: 10.1038/ejcn.2008.14]

    Reason for exclusion Wrong outcomes

17. Genova M.; Atanasova B.; Ivanova I.; Todorova K.; Svinarov D.. Trace Elements and Vitamin D in Gestational Diabetes. Acta Medica Bulgarica 2018;45(1):45-49. [DOI: http://dx.doi.org/10.2478/amb-2018-0009]

    Reason for exclusion Wrong study design

18. Hossein-Nezhad A.; Maghbooli J.; Arzaghi S.M.; Shafaei A.; Rahmani M.; Larijani B.. Relationship between vitamin D deficiency and gestational diabetes mellitus. Iranian Journal of Diabetes and Lipid Disorders 2006;5(3):E25. [DOI:]

    Reason for exclusion Not English

19. Inancli, I. S.; Yayci, E.; Atacag, T.; Uncu, M.. Is maternal Vitamin D associated with gestational diabetes mellitus in pregnant women in Cyprus? Clinical and Experimental Obstetrics & Gynecology 2016;43(6):840-843. [DOI: 10.12891/ceog3152.2016]

    Reason for exclusion Wrong outcomes

20. Joergensen JS.; Lamont RF.; Torloni MR.. Vitamin D and gestational diabetes: an update.. Current opinion in clinical nutrition and metabolic care 2014;17(4):360-7. [DOI: 10.1097/MCO.0000000000000064]

    Reason for exclusion Wrong study design

21. Karamali, M; Asemi, Z; Ahmadi-Dastjerdi, M; Esmaillzadeh, A. Calcium plus vitamin D supplementation affects pregnancy outcomes in gestational diabetes: randomized, double-blind, placebo-controlled trial. 2016;19(1):156-163. [DOI: 10.1017/S1368980015000609]

    Reason for exclusion Wrong outcomes

22. Khadgawat, R. Safety and efficacy of vitamin d supplementation during pregnancy: a randomized double blind control. 2018;143:205-. [DOI: 10.1002/ijgo.12582]

    Reason for exclusion Wrong outcomes

23. Khadgawat R.; Kachhawa G.; Kulshreshtha V.; Gupta T.; Sreenivas V.; Gupta N.. Vitamin D supplementation in pregnancy: A randomized double-blind controlled trial. Calcified Tissue International 2019;104(Supplement 1):S44. [DOI: http://dx.doi.org/10.1007/s00223-019-00544-x]

    Reason for exclusion Conference abstract with no new data

24. Khorasani ZM.; Bonakdaran S.; Rafieie HP.. The relationship between vitamin D deficiency and insulin resistance in pregnant women with gestational diabetes.. Current diabetes reviews 2018. [DOI: 10.2174/1573399814666181102100816]

    Reason for exclusion Wrong outcomes

25. Kim S.-K.; Kim K.-S.; Park S.W.; Cho Y.-W.. Vitamin D deficiency is associated with higher risk of postpartum glucose intolerance in women with gestational diabetes mellitus. Diabetes Research and Clinical Practice 2014;106(SUPPL. 1):S76-S77. [DOI:]

    Reason for exclusion Conference abstract with no new data

26. Kintiraki, Evangelia; Goulis, Dimitrios G. Gestational diabetes mellitus: Multi-disciplinary treatment approaches. Metabolism 2018;86:91-101. [DOI:]

    Reason for exclusion Wrong study design

27. Lacroix M.; Battista MC.; Doyon M.; Houde G.; Ménard J.; Ardilouze JL.; Hivert MF.; Perron P.. Lower vitamin D levels at first trimester are associated with higher risk of developing gestational diabetes mellitus.. Acta diabetologica 2014;51(4):609-16. [DOI: 10.1007/s00592-014-0564-4]

    Reason for exclusion Wrong outcomes

28. Lau SL.; Gunton JE.; Athayde NP.; Byth K.; Cheung NW.. Serum 25-hydroxyvitamin D and glycated haemoglobin levels in women with gestational diabetes mellitus.. The Medical journal of Australia 2011;194(7):334-7. [DOI:]

    Reason for exclusion Wrong outcomes

29. Li, Q; Xing, B. Vitamin D3-Supplemented Yogurt Drink Improves Insulin Resistance and Lipid Profiles in Women with Gestational Diabetes Mellitus: a Randomized Double Blinded Clinical Trial. 2016;68(4):285-290. [DOI: 10.1159/000447433]

    Reason for exclusion Wrong outcomes

30. Lim, Sun-Young; Yoo, Hyun-Jung; Kim, Ae-Lan; Oh, Jeong-Ah; Kim, Hun-Sung; Choi, Yoon-Hee; Cho, Jae-Hyoung; Lee, Jin-Hee; Yoon, Kun-Ho. Nutritional intake of pregnant women with gestational diabetes or type 2 diabetes mellitus. Clinical nutrition research 2013;2(2):81-90. [DOI:]

    Reason for exclusion Wrong outcomes

31. Liu F.; Hou W.; Tang J.; Lu J.; He R.; Jia W.. Serum vitamin D levels in gestational diabetes mellitus patients and its relationship with islet cell function. Diabetes 2016;65(Supplement 1):A589. [DOI: http://dx.doi.org/10.2337/db16-2228-2366]

    Reason for exclusion 32. Wrong outcomes

33. Macones GA.; Norman S.; Hopeman M.; McCartney SA.. Discussion: 'vitamin D deficiency in pregnancy and gestational diabetes,' by Burris et al.. American journal of obstetrics and gynecology 2012;207(3):e1-3. [DOI: 10.1016/j.ajog.2012.07.019]

    Reason for exclusion Wrong outcomes

34. Mcmanus R.M.; Giroux I.; Summers K.; Cauchi L.; Devrijer B.; Thompson A.. Gestational diabetes, vitamin D intake and vitamin D insufficiency. Diabetes 2010;((Mcmanus, Giroux, Summers, Cauchi, Devrijer, Thompson) LondonONCanada). [DOI:]

    Reason for exclusion Wrong outcomes

35. Mutlu N.; Esra H.; Begum A.; Fatma D.; Arzu Y.; Yalcin H.; Fatih K.; Selahattin K.. Relation of maternal vitamin D status with gestational diabetes mellitus and perinatal outcome.. African health sciences 2015;15(2):523-31. [DOI: 10.4314/ahs.v15i2.27]

    Reason for exclusion Wrong outcomes

36. Napartivaumnuay N.; Niramitmahapanya S.; Deerochanawong C.; Suthornthepavarakul T.; Sarinnapakorn V.; Jaruyawongs P.. Maternal 25 hydroxyvitamin D level and its correlation in Thai gestational diabetes patients. Journal of the Medical Association of Thailand 2013;96(SUPPL.3):69-76. [DOI:]

    Reason for exclusion Wrong outcomes

37. NCT01992133. Effects of Vitamin D Supplementation on Glucose Metabolism in Women With Former Gestational Diabetes Mellitus. 2013. [DOI:]

    Reason for exclusion Clinical trial registration

38. NCT03138616. The Impact of Vitamin D Supplementation on Glucose Metabolism in Chinese Women With Former Gestational Diabetes Mellitus. 2017. [DOI:]

    Reason for exclusion Clinical trial registration

39. NTR3158. Vitamin D supplementation in Gestational Diabetes Mellitus. 2011. [DOI:]

    Reason for exclusion Clinical trial registration

40. Ojo O.; Weldon SM.; Thompson T.; Vargo EJ.. The Effect of Vitamin D Supplementation on Glycaemic Control in Women with Gestational Diabetes Mellitus: A Systematic Review and Meta-Analysis of Randomised Controlled Trials.. International journal of environmental research and public health 2019;16(10). [DOI: 10.3390/ijerph16101716]

    Reason for exclusion Wrong study design

41. Palacios, C.; De-Regil, L. M.; Lombardo, L. K.; Pena-Rosas, J. P.. Vitamin D supplementation during pregnancy: Updated meta-analysis on maternal outcomes. Journal of Steroid Biochemistry and Molecular Biology 2016;164:148-155. [DOI: 10.1016/j.jsbmb.2016.02.008]

    Reason for exclusion Duplicate

42. Parlea, L.; Bromberg, I. L.; Feig, D. S.; Vieth, R.; Merman, E.; Lipscombe, L. L.. Association between serum 25-hydroxyvitamin D in early pregnancy and risk of gestational diabetes mellitus. Diabetic Medicine 2012;29(7):E25-E32. [DOI: 10.1111/j.1464-5491.2011.03550.x]

    Reason for exclusion Wrong outcomes

43. Pendek R.; Nair K.; San Lim S.; Ananda V.; Tan A.T.B.; Chan S.P.. Vitamin D status and risk factors for progression to glucose intolerance in women with previous gestational diabetes in Malaysia. Diabetes 2011;60(SUPPL. 1):A663. [DOI: http://dx.doi.org/10.2337/db11-2432-2532]

Reason for exclusion Wrong outcomes

44. Perez-Ferre N.; Torrejon MJ.; Fuentes M.; Fernandez MD.; Ramos A.; Bordiu E.; del Valle L.; Rubio MA.; Bedia AR.; Montañez C.; Calle-Pascual AL. Association of low serum 25-hydroxyvitamin D levels in pregnancy with glucose homeostasis and obstetric and newborn outcomes. Endocrine practice : official journal of the American College of Endocrinology and the American Association of Clinical Endocrinologists ;18(5):676-84. [DOI:]

Reason for exclusion Wrong outcomes

45. Pleskačová, Anna; Bartáková, Vendula; Pácal, Lukáš; Kuricová, Katarína; Bělobrádková, Jana; Tomandl, Josef; Kaňková, Kateřina. Vitamin D status in women with gestational diabetes mellitus during pregnancy and postpartum. BioMed research international 2015;2015. [DOI:]

Reason for exclusion Wrong outcomes

46. Poel, Y. H. M.; Hummel, P.; Lips, P.; Stam, F.; van der Ploeg, T.; Simsek, S.. Vitamin D and gestational diabetes: A systematic review and meta-analysis. European Journal of Internal Medicine 2012;23(5):465-469. [DOI: 10.1016/j.ejim.2012.01.007]

Reason for exclusion Wrong study design

47. Rodrigues, Meline Rossetto Kron; Lima, Silvana Andréa Molina; Mazeto, Glaucia Maria Ferreira da Silvia; Calderon, Iracema Mattos Paranhos; Magalhães, Claudia Garcia; Ferraz, Guilherme Augusto Rago; Molina, Ana Claúdia; Costa, Roberto Antônio de Araújo; Nogueira, Vania dos Santos Nunes; Rudge, Marilza Vieira Cunha. Efficacy of vitamin D supplementation in gestational diabetes mellitus: Systematic review and meta-analysis of randomized trials. PLOS ONE 2019;14(3):e0213006. [DOI:]

Reason for exclusion Wrong study design

48. Rutkowska, J.; Bandurska-Stankiewicz, E.; Wiatr-Bykowska, D.; Myszka-Podgorska, K.; Kuglarz, E.; Matuszewski, W.. Vitamin D deficiency in women with gestational diabetes mellittus. Clinical Diabetology 2016;5(2):57-61. [DOI: 10.5603/dk.2016.0010]

Reason for exclusion 49. Duplicate

50. Rutkowska J.; Bandurska-Stankiewicz E.; Wiatr-Bykowska D.; Myszka-Podgorska K.; Kuglarz E.; Matuszewski W.. Vitamin D deficiency in women with gestational diabetes mellitus. Clinical Diabetology 2016;5(2):57-61. [DOI: http://dx.doi.org/10.5603/DK.2016.0010]

Reason for exclusion 51. Wrong outcomes

Sahin E.; Col Madendag I.; Sahin ME.; Madendag Y.; Acmaz G.; Muderris II.. Effect of vitamin D deficiency on the 75 g oral glucose tolerance test screening and insulin resistance.. Gynecological endocrinology : the official journal of the International Society of Gynecological Endocrinology 2019;35(6):535-538. [DOI: 10.1080/09513590.2018.1554038]

Reason for exclusion Wrong outcomes

52. Soheilykhah, S.; Mojibian, M.; Rashidi, M.; Rahimi-Saghand, S.; Jafari, F.. Maternal Vitamin D Status in Gestational Diabetes Mellitus. Nutrition in Clinical Practice 2010;25(5):524-527. [DOI: 10.1177/0884533610379851]

Reason for exclusion Wrong study design

53. Triunfo S.; Lanzone A.; Lindqvist PG.. Low maternal circulating levels of vitamin D as potential determinant in the development of gestational diabetes mellitus.. Journal of endocrinological investigation 2017;40(10):1049-1059. [DOI: 10.1007/s40618-017-0696-9]

Reason for exclusion Wrong study design

54. von Websky, K.; Hasan, A. A.; Reichetzeder, C.; Tsuprykov, O.; Hocher, B.. Impact of vitamin D on pregnancy-related disorders and on offspring outcome. Journal of Steroid Biochemistry and Molecular Biology 2018;180:51-64. [DOI: 10.1016/j.jsbmb.2017.11.008]

Reason for exclusion Wrong study design

55. Walsh JM.; McGowan CA.; Kilbane M.; McKenna MJ.; McAuliffe FM.. The relationship between maternal and fetal vitamin D, insulin resistance, and fetal growth.. Reproductive sciences (Thousand Oaks, Calif.) 2013;20(5):536-41. [DOI: 10.1177/1933719112459222]

Reason for exclusion Wrong outcomes

56. Walsh, M.; Barebring, L.; Augustin, H.. Avoiding maternal vitamin D deficiency may lower blood glucose in pregnancy. Journal of Steroid Biochemistry and Molecular Biology 2019;186:117-121. [DOI: 10.1016/j.jsbmb.2018.10.003]

Reason for exclusion Wrong outcomes

57. Wang O.; Nie M.; Hu YY.; Zhang K.; Li W.; Ping F.; Liu JT.; Chen LM.; Xing XP.. Association between vitamin D insufficiency and the risk for gestational diabetes mellitus in pregnant Chinese women.. Biomedical and environmental sciences : BES 2012;25(4):399-406. [DOI: 10.3967/0895-3988.2012.04.004]

Reason for exclusion Wrong outcomes

58. Xu C.; Ma HH.; Wang Y.. Maternal Early Pregnancy Plasma Concentration of 25-Hydroxyvitamin D and Risk of Gestational Diabetes Mellitus.. Calcified tissue international 2018;102(3):280-286. [DOI: 10.1007/s00223-017-0346-4]

Reason for exclusion Wrong outcomes

59. Yap, C; Cheung, NW; Gunton, JE; Athayde, N; Munns, CF; Duke, A; McLean, M. Vitamin D supplementation and the effects on glucose metabolism during pregnancy: a randomized controlled trial. 2014;37(7):1837-1844. [DOI: 10.2337/dc14-0155]

Reason for exclusion Wrong outcomes

60. Zhang, M. X.; Pan, G. T.; Guo, J. F.; Li, B. Y.; Qin, L. Q.; Zhang, Z. L.. Vitamin D Deficiency Increases the Risk of Gestational Diabetes Mellitus: A Meta-Analysis of Observational Studies. Nutrients 2015;7(10):8366-8375. [DOI: 10.3390/nu7105398]

Reason for exclusion Wrong study design

61. Zhang Y.; Gong Y.; Xue H.; Xiong J.; Cheng G.. Vitamin D and gestational diabetes mellitus: a systematic review based on data free of Hawthorne effect.. BJOG : an international journal of obstetrics and gynaecology 2018;125(7):784-793. [DOI: 10.1111/1471-0528.15060]

Reason for exclusion Wrong study design

62. Zuhur, S. S.; Erol, R. S.; Kuzu, I.; Altuntas, Y.. The relationship between low maternal serum 25-hydroxyvitamin D levels and gestational diabetes mellitus according to the severity of 25-hydroxyvitamin D deficiency. Clinics 2013;68(5):658-664. [DOI: 10.6061/clinics/2013(05)13]

Reason for exclusion Wrong outcomes

Table S2: Baseline maternal mean (SD) BMI in the six included studies on vitamin D in gestational diabetes and markers of type 2 diabetes development

**Study (design)**		N (%)	Median (25th, 75th percentiles) or Mean±SD	p-value difference within group	N	Median (25th, 75th percentiles) or Mean±SD	p-value difference within group	Between group P-value
[22] Kramer et al.	Deficiency	161 (33)	27.8 (23.9-32.3)			-	-	-
(cohort)	Insufficiency	178 (36)	25.3 (23.4-28.9)			-	-	-
	Sufficiency	155 (31)	24.5 (22.1-27.6)			-	-	<.001
[18] Shaat et al.	Deficiency	198 (53)	26.2 ± 5.6			-	-	-
(cross-sectional)	Insufficiency	125 (33)	24.7 ± 5.0			-	-	-
	Sufficiency	53 (13)	23.0 ± 3.1			-	-	<.001
		Cases			Controls			
[19]Tänczer et al. (case-control)		87	25.9±5.9		45	24.3±4.4		.119
		Intervention group			Control group			
[21] Yeow et al. (RCT)	Baseline	13	26.5 (21.9-30.7)		13	28.8 (25.6-40.0)		0.397
[17] Mozaffari-Khosravi et al. (RCT)	Baseline	24	28.9 ± 4.8		21	27.9 ± 3.6		0.4
[20]Valizadeh et al. (RCT)	Baseline	42	27.6 ± 3.9		42	27.6 ± 3.8		.94

SD: standard deviation. BMI: body mass index (Kg/m^2)

Table S3: Synthesis of results with direction of associations by outcome among the three included RCTs on vitamin D in gestational diabetes and markers of type 2 diabetes development

Outcomes	Study	Direction of associations
**Insulin sensitivity**		
HOMA-S	Mozaffari-Khosravi et al.	(+)
QUICKI	Mozaffari-Khosravi et al.	(+)
	Yeow et al.	(-)
OGIS	Yeow et al.	no
BIGTT-S	Yeow et al.	no
**Insulin resistance**		
HOMA-IR	Mozaffari-Khosravi et al.	(-)
	Valizadeh et al.	no
Fasting insulin	Valizadeh et al.	no
	Yeow et al.	(+)
Fasting C-peptide	Yeow et al.	no
**Beta-cell function**		
HOMA-B	Mozaffari-Khosravi et al.	no
Disposition index	Yeow et al.	no
AUCinsulin (pmol/l)	Yeow et al.	no
AUCcp (ng/ml)	Yeow et al.	no
IGI_{60} (pmol/ mmol)	Yeow et al.	no
BIGTT-AIR	Yeow et al.	no
**Glucose measurements**		
FBG	Mozaffari-Khosravi et al.	no
FPG	Valizadeh et al.	no
	Yeow et al.	no
30-minute 75g OGTT	Yeow et al.	no
2h 75g OGTT	Mozaffari-Khosravi et al.	no
	Valizadeh et al.	no
AUCglucose (mmol/L)	Yeow et al.	no
**Glycated hemoglobin**		
HbA1c	Mozaffari-Khosravi et al.	no
	Valizadeh et al.	no
	Yeow et al.	no
% HbA1c	Mozaffari-Khosravi et al.	no
	Valizadeh et al.	no
**Diabetes**	[**]Shaat et al.	no
	[***]Valizadeh et al.	no

No: no statistically significant association; (+) direct/positive association; (-) inverse association
HOMA-IR: homeostasis model assessment of insulin resistance; QUICKI: quantitative insulin sensitivity check index; ISSI-2: The insulin secretion-sensitivity index-2; OGTT: oral glucose tolerance test; HOMA(2)-S: homeostasis model assessment insulin resistance; HOMA(2)-B: homeostasis model assessment beta-cell function; FBG: fasting blood glucose; FPG: fasting plasma glucose; HbA1c: Hemoglobin A1c; OGIS: oral glucose insulin sensitivity index; AUC: area under the curve; AUCcp: area under the curve of C-peptide; IGI60: insulinogenic index calculated at 60 minutes; BIGTT: pancreatic beta-cell function, insulin sensitivity and glucose tolerance test; BIGTT-S: BIGTT with insulin sensitivity; BIGTT-AIR: BIGTT with acute insulin response
[*]25(OH)D sufficiency as reference group

[**]based on the WHO 1999 criteria: fasting 2 hours 75g OGTT: ≥ 140 mg/dL (7.8 mmol/L)
[***]Dysglycemia was defined as the development impaired fasting glucose (IFG) or impaired glucose tolerance (IGT) or type 2 diabetes in subjects as measured by the postpartum tests. IFG was defined by FPG levels of 100 to 125 mg/dL, IGT by 2-hPLG levels of 140 to 199 mg/dL, and type 2 diabetes by FPG levels ≥ 126 or 2-PLG levels ≥ 200 mg/dL

Table S4: Summary of ROBINS-I and Cochrane Risk of Bias assessment

Risk of bias								
**Observational studies – ROBINS - I**								
Study	**Domain 1** Confounding	**Domain 2** Selection of participants	**Domain 3** Classification of interventions	**Domain 4** Departure from intended interventions	**Domain 5** Missing data	**Domain 6** Measurements of outcome	**Domain 7** Selection of reported results	**Overall judgement**
**Kramer (2014)**	Serious	Low	Low	NI	Low	Moderate	Moderate	Serious
**Shaat (2017)**	Critical	Low	Low	NI	Low	Moderate	Moderate	Critical
**Tänczer (2017)**	Critical	Low	Low	NI	Critical	Moderate	Moderate	Critical
**Randomized controlled trials – Cochranes Risk of Bias tool**								
Study	Sequence Generation	Allocation concealment	Blinding of participants	Blinding og outcome assessors	Incomplete outcome data	Selective outcome reporting	Other sources of bias	
Mozaffari-Khosravi 2017	Unclear	Unclear	Low	Low	Unclear	Low	Low	-
Valizadeh 2016	Low	Low	Low	Low	Low	Low	Low	-
Yeow 2015	Unclear	Low	Low	Low	Low	Low	Low	-

The role of vitamin D in the development of diabetes post gestational diabetes mellitus: a systematic literature review

A.C. Keller, C. Varela, R. Dangol, N.P. Damm, B.L. Heitmann, M. N. Händel
